# Heterogeneity of Treatment Effects in Internet- and Mobile-Based Interventions for Depression: A Systematic Review and Meta-Analysis

**DOI:** 10.1001/jamanetworkopen.2024.23241

**Published:** 2024-07-18

**Authors:** Yannik Terhorst, Tim Kaiser, Eva-Lotta Brakemeier, Isaac Moshe, Paula Philippi, Pim Cuijpers, Harald Baumeister, Lasse Bosse Sander

**Affiliations:** 1 Department of Clinical Psychology and Psychotherapy, Institute of Psychology and Education, University Ulm, Ulm, Germany; 2 Department of Psychology, Ludwig Maximilian University of Munich, Munich, Germany; 3 Methods and Evaluation/Quality Assurance, Freie Universität Berlin, Berlin, Germany; 4 Department of Clinical Psychology and Psychotherapy, University Greifswald, Greifswald, Germany; 5 Department of Psychology and Logopedics, Faculty of Medicine, University of Helsinki, Helsinki, Finland; 6 Department of Clinical Child and Adolescent Psychology and Psychotherapy, Bergische Universität Wuppertal, Wuppertal, Germany; 7 Department of Clinical, Neuro-, and Developmental Psychology, Amsterdam Public Health Research Institute, Vrije Universiteit Amsterdam, Amsterdam, the Netherlands; 8 Medical Psychology and Medical Sociology, Faculty of Medicine, University of Freiburg, Freiburg im Breisgau, Germany

## Abstract

**Importance:**

While the effects of internet- and mobile-based interventions (IMIs) for depression have been extensively studied, no systematic evidence is available regarding the heterogeneity of treatment effects (HTEs), indicating to what extent patient-by-treatment interactions exist and personalized treatment models might be necessary.

**Objective:**

To investigate the HTEs in IMIs for depression as well as their efficacy and effectiveness.

**Data Sources:**

A systematic search in Embase, MEDLINE, Central, and PsycINFO for randomized clinical trials and supplementary reference searches was conducted on October 13, 2019, and updated March 25, 2022. The search string included various terms related to digital psychotherapy, depression, and randomized clinical trials.

**Study Selection:**

Titles, abstracts, and full texts were reviewed by 2 independent researchers. Studies of all populations with at least 1 intervention group receiving an IMI for depression and at least 1 control group were eligible, if they assessed depression severity as a primary outcome and followed a randomized clinical trial (RCT) design.

**Data Extraction and Synthesis:**

This study followed the Preferred Reporting Items for Systematic Reviews and Meta-Analyses reporting guidelines. Risk of bias was evaluated using the Cochrane Risk of Bias Tool. HTE was investigated using logarithmic variance ratios (lnVR) and effect sizes using Hedges *g*. Three-level bayesian meta-regressions were conducted.

**Main Outcomes and Measures:**

Heterogeneity of treatment effects was the primary outcome of this study; magnitudes of treatment effect sizes were the secondary outcome. Depression severity was measured by different self-report and clinician-rated scales in the included RCTs.

**Results:**

The systematic review of 102 trials included 19 758 participants (mean [SD] age, 39.9 [10.58] years) with moderate depression severity (mean [SD] in Patient Health Questionnaire–9 score, 12.81 [2.93]). No evidence for HTE in IMIs was found (lnVR = −0.02; 95% credible interval [CrI], −0.07 to 0.03). However, HTE was higher in more severe depression levels (β̂ = 0.04; 95% CrI, 0.01 to 0.07). The effect size of IMI was medium (*g* = −0.56; 95% CrI, −0.46 to −0.66). An interaction effect between guidance and baseline severity was found (β̂ = −0.24, 95% CrI, −0.03 to −0.46).

**Conclusions and Relevance:**

In this systematic review and meta-analysis of RCTs, no evidence for increased patient-by-treatment interaction in IMIs among patients with subthreshold to mild depression was found. Guidance did not increase effect sizes in this subgroup. However, the association of baseline severity with HTE and its interaction with guidance indicates a more sensitive, guided, digital precision approach would benefit individuals with more severe symptoms. Future research in this population is needed to explore personalization strategies and fully exploit the potential of IMI.

## Key Points

**Question:**

Is there evidence from randomized clinical trials (RCTs) that patients respond differently to internet- and mobile-based interventions (IMIs) for depression?

**Findings:**

In this meta-analysis of 102 RCTs involving 19 758 participants, clinically relevant effect sizes for unguided IMIs in patients with subthreshold to mild depression without evidence for substantial patient-by-treatment interaction was found. In contrast, heterogeneity of treatment effects and moderating effects of guidance increased with baseline depression severity.

**Meaning:**

These findings suggest that moderate improvements in subthreshold to mild depression can be reasonably expected from unguided IMIs, but individuals with more severe depression could respond differently, indicating the need for digital precision psychotherapy and future research in this subgroup.

## Teaser

This systematic review and meta-analysis examines whether internet- and mobile-based interventions (IMIs) for depression have heterogeneous treatment effects.

## Introduction

Major depression constitutes one of the leading causes of disease burden worldwide, with a global prevalence of 3.2% (95% CI, 2.7%-3.7%) and 49.4 (95% CI, 33.6-68.7) million disability-adjusted life-years worldwide in 2020.^1,2,3^ Besides the high personal burden for affected individuals, depression is related to high economic costs and increased risk for chronic diseases and mortality, further highlighting the need for effective depression treatment.^4,5,6,7^ In addition to pharmacological therapy^8^ and face-to-face psychotherapy for depression,^9,10^ internet- and mobile-based interventions (IMIs) have been frequently studied in the last 2 decades.^11,12^

IMIs transfer face-to-face psychotherapeutic approaches into the virtual space providing time- and location-independent access to treatment.^11,12,13^ Typically, they present web-based programs with segmented modules, often organized into weekly sessions (eg, psychoeducation, problem-solving, activity activation).^11^ More recently, mobile health applications have also been studied.^11,14^ Besides their technical setup, IMIs primarily hinge on cognitive behavioral therapy (CBT) and differ in the level of human support, ranging from unguided approaches (ie, completely standardized treatment protocols without human involvement) to guided interventions with human therapeutic guidance (eg, e-coach feedback on exercises or recommendations for actions).^11,15,16^

A recent meta-analysis of randomized clinical trials (RCTs)^11^ indicated an overall effect size (ES) of IMIs of *g* = 0.52 (95% CI, 0.43-0.60) for depression severity. Subgroup analysis in a smaller set of pragmatic effectiveness trials also showed effectiveness, with *g* = 0.30 (95% CI, 0.15-0.45).^11^ The few studies comparing IMI with traditional face-to-face psychotherapy showed comparable effects.^8,17,18,19,20^ This evidence strongly suggests that IMIs are an effective treatment for depression.^11^ However, whether IMIs have consistent effects for all individuals or whether there is substantial heterogeneity of treatment effects (HTE) is an open question, potentially with implications for precision psychotherapy.^11,12,16,21^

HTE can be systematically investigated by analyzing RCTs using the following rationale^22,23,24^: in an RCT, randomly assigning participants to a control (CG) or an intervention group (IG) eliminates casual differences between the groups. Hence, the severity means and variance in the IG and the CG are equivalent after randomization. In the case of an effective intervention, the means in the IG and the CG are different after treatment. Similarly, the variance ratio can be investigated. If there is more variance in the individual responses in the IG compared with the CG, this provides evidence for the presence of subgroups responding differently to the intervention (eAppendix 1 in Supplement 1). Hence, a meta-analysis on the variance ratios between IG and CG reported in RCTs can evaluate whether higher individual responses and substantial HTE are systematically occurring for a treatment type compared with control conditions. A greater extent of unequal variance would imply stronger evidence for the presence of meaningful patient-by-treatment interaction.^22,23,24^

HTE in meta-analyses was initially studied in other health conditions^24,25,26^ and, more recently, in pharmacological treatments and face-to-face therapy for depression.^23,27^ In depression treatment, antidepressants do not show substantial HTE, indicating that they are an effective treatment option without substantial variations in effectiveness compared with treatment as usual or other control groups.^23^ In contrast, face-to-face psychotherapy shows HTE, indicating the presence of subgroups that respond particularly well to it and to specific treatment types (eg, behavioral activation therapy or cognitive-behavioral therapy).^27^ Hence, optimizing face-to-face psychotherapy by identifying well-responding subgroups and tailoring (eg, by selecting treatment type or taking known moderators into account, like depression episode number, duration, and severity) can mark an important step toward improved psychotherapy.^27,28,29,30^

Given that IMIs usually transfer existing face-to-face psychotherapeutic approaches to a digital platform, the question arises whether the evidence for systematic patient-by-treatment interactions transfers to IMIs or is eliminated (eg, through the high standardization of treatment protocols in IMIs). In the case of equivalent variances between IG and CG, we could reasonably assume the average meta-analytical ES of IMIs for the individual. In contrast, substantial HTE would provide evidence that only certain subgroups respond to IMIs, indicating a potential need for digital precision psychotherapy.

In this study, we systematically review and analyze RCTs on IMIs for depression to provide evidence of whether substantial HTE exists. Additionally, we extend previous findings on the ES of IMIs for depression with a primary interest in the role of guidance, baseline severity, and evidence for effectiveness beyond highly controlled laboratory settings.^11,12,31,32^

## Methods

### Study Design and Search Strategy

The present study is a systematic review and meta-analysis of the HTE of IMIs for depression and extends a previous analysis on the efficacy and effectiveness of IMIs for depression.^11^ We searched the literature databases Embase, MEDLINE, Central, and PsycINFO for relevant articles. The original search was conducted on October 13, 2019, and updated for the present study on March 25, 2022. The complete search string can be found in eAppendix 2 in Supplement 1. Second, we reviewed the reference lists of previous systematic reviews.^11^ Lastly, we performed a backward search in the reference list of all included studies. All procedures have been registered in the open science framework.^33^ We followed the Preferred Reporting Items for Systematic Reviews and Meta-analyses (PRISMA)^34^ guidelines for reporting systematic reviews in the present study.

### Inclusion Criteria and Data Extraction

We applied the following predefined population, intervention, comparator, outcome, and study design (PICOS) inclusion criteria:

- Population: studies with participants of all ages with depressive symptoms were included, and all genders, nationalities, and cultural backgrounds were eligible;
- Intervention: studies needed to apply at least 1 IMI for depression (ie, computer-, online-, internet-, web-, or smartphone-based intervention), and the IMI could be provided online or offline;
- Comparison: IG(s) needed to be compared against at least 1 CG, and both inactive (eg, waiting list CG) and active (eg, treatment as usual) CGs were eligible;
- Outcomes: depression severity must have been included and measured by a validated self- or clinician-rated depression scale;
- Study design: to be included, studies needed to follow an RCT design and all studies needed to be approved by an institutional review board or ethics committee and have obtained informed consent from their participants.

For the coding and data extraction, 2 independent researchers assessed each included study (2 of Y.T., L.B.S., I.M., and P.P.). Participant characteristics (eg, mean age), design aspects (eg, type of control group), intervention details (eg, guidance), and method features (eg, missing data handling) were extracted. All disagreements in data extraction were resolved in discussion, and the required data (eg, variance ratios and ESs) for analyses could be obtained for all included studies.

### Risk of Bias

We used the Cochrane Risk of Bias Tool I to assess study quality.^35^ Accordingly, the risk of bias was rated as low, unclear, or high on the 7 sources of bias: (1) random sequence generation, (2) allocation concealment, (3) blinding of participants and personnel, (4) blinding of outcome assessment, (5) incomplete outcome data, (6) selective reporting, and (7) other. In principle, blinding of participants and personnel in psychotherapeutic research is not feasible.^36^ Hence, we have rated all self-report instruments (answered by unblinded participants) in the domain of blinding of outcome assessors as having high risk of bias.

### Statistical Analysis

#### HTE of IMIs for Depression

We followed the procedures of previous analyses in the context of pharmacological and psychotherapeutic interventions for depression and conducted a 3-level bayesian random-effects meta-regression.^23,27,37^ The primary outcome was the logarithmic variance ratio (lnVR) of depression severity between IG and CG (level 1) at post assessment while allowing for differences between outcomes within a study (level 2, eg, multiple depression outcomes or CGs) and between studies (level 3). Hence, the level 1 estimate provides an estimate of the extent the variances in the IG and CG differ. A positive estimate indicates higher variance in the IG compared with CG and would provide meta-analytical evidence for substantial patient-by-treatment interaction and patients responding differently to IMIs compared with control conditions.^22,23,27^ For further details on model parameters, see eMethods 1 in Supplement 1. To avoid bias through a possible mean-variance relationship, we controlled for differences in mean scores by including the logarithm of the posttreatment severity mean ratio (lnER) from the IG to the CG.^22,23,27^ We selected weak priors in all analyses (eMethods 2 in Supplement 1).

#### HTE Sensitivity and Subgroup Analysis

We conducted sensitivity and subgroup analyses to investigate the role of various design and study characteristics (ie, effectiveness and efficacy settings and control types), intervention characteristics (ie, guidance, therapeutic background, and delivery format), participant characteristics (ie, age, gender, and baseline severity), potential long-term HTE, year of publication, assessment time, and risk of bias. eMethods 3 in Supplement 1 includes coding and analysis details. Subgroup analyses were only conducted for subgroups with at least 10 studies.^27^

#### Secondary Analysis on ESs of IMI for Depression

Analogous to the HTE analysis, we conducted secondary analyses on the ESs of IMI on depression severity (Hedges *g*) using bayesian 3-level meta-regression (eMethods 4 in Supplement 1). Analyses included subgroup and moderation analyses (eg, guidance, baseline severity, or setting) as outlined in eMethods 3 in Supplement 1.

#### Software

The statistical software R version 4.2.2 (R Project for Statistical Computing) was used for all analyses. The R packages rstan^38^ and brms^39^ were used as the core package for the analysis. eAppendix 3 in Supplement 1 provides an overview of all packages. Analysis code and used data are freely available.^40^

## Results

### Study Characteristics

We included 102 trials^17,18,19,41,42,43,44,45,46,47,48,49,50,51,52,53,54,55,56,57,58,59,60,61,62,63,64,65,66,67,68,69,70,71,72,73,74,75,76,77,78,79,80,81,82,83,84,85,86,87,88,89,90,91,92,93,94,95,96,97,98,99,100,101,102,103,104,105,106,107,108,109,110,111,112,113,114,115,116,117,118,119,120,121,122,123,124,125,126,127,128,129,130,131,132,133,134,135,136,137,138,139,140,141,142,143,144^ comprising a total of 19 758 participants with a mean (SD) age of 39.9 (10.58) years, a mean (SD) percentage of female participants of 69.13% (12.22), and moderate depression severity across the studies (mean [SD] Patient Health Questionnaire–9 score, 12.81 [2.93]). The PRISMA flowchart and more information about the dataset can be found in eAppendices 4 and 5 in Supplement 1 and the eTable in Supplement 2. The included trials were predominantly conducted in Europe (61 [59.80%]), followed by Canada and the United States (17 [16.67%]), Australia and New Zealand (16 [15.69%]), and Asia (8 [7.84%]). Most studies investigated the efficacy of IMIs (78 [76.47%]) and compared IMIs with waiting list CGs (47 [44.76%]), followed by treatment as usual (27 [25.71%]), attention control (24 [22.86%]), face-to-face psychotherapy (6 [5.71%]), and 1 other (0.98%).^84^ IMIs were based on cognitive behavioral therapy (71 [68.27%]) most frequently (third-wave therapy: 14 [13.46%]; problem-solving-therapy: 8 [7.69%]; psychodynamic therapy: 1 [0.98%]; life review therapy: 1 [0.98%]; other (eg, combined approaches): 9, [8.65%]). Therapeutic support by humans was provided in 56 studies (51.85%), while technical guidance was used in 27 (25.00%), and no guidance in 25 (23.15%). Internet-based interventions were most frequent (88 [86.27%]), followed by smartphone app–based interventions (6 [5.88%]), computer-based interventions (5 [4.90%]), and interventions combining internet and smartphone app (3 [2.94%]).

### Risk of Bias and Study Quality

Most studies (94 [91.18%]) showed a low risk of bias in sequence generation (unclear: 5 [4.9%]; high: 3 [2.94%]). Allocation concealment was low in 79 studies (77.45%), unclear in 20 (19.61%), and high in 3 (2.94%). Blinding of participants was the domain with the highest risk of bias: 1 study was rated as low (0.98%), 17 (16.67%) as unclear, and 84 (82.35%) as high. Outcome assessors were not masked in 65 studies (63.73%), indicating a high risk of bias; risk of bias was unclear in 22 (21.57%) and low in 15 (14.71%). Of the 93 included studies (91.12%) that followed an intention-to-treat analysis, 70 (75.27%) applied adequate missing data handling. Overall bias due to incomplete outcome data was rated as low in 73 (71.47%), unclear in 3 (2.94%), and high in 26 (25.49%). Regarding selective reporting, only 1 study (0.98%) was rated as high, while 75 (73.53%) were rated low and 26 (25.49%) unclear. Analogous to sequence generation, other sources of bias were mostly low (94 [92.16%]; unclear: 5 [4.9%]; high: 3 [2.94%]) (Figure). Study-wise risk of bias ratings are reported in eAppendix 6 in Supplement 1. Sensitivity analysis on study quality did not affect results meaningfully (eAppendix 7 in Supplement 1).

**Figure.**
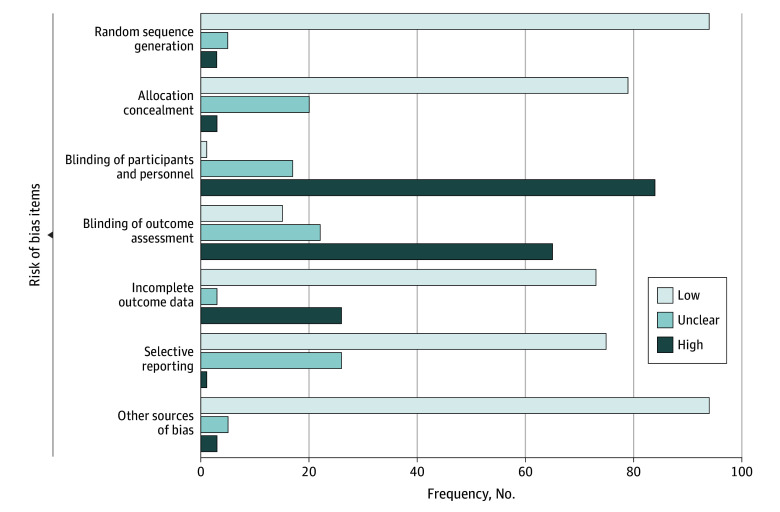
Summary of Risk of Bias Ratings According to the Cochrane Risk of Bias I Tool

### HTE in IMI for Depression

The 102 included studies provided 153 comparisons between IG and CG at post treatment. The primary analysis of HTE in IMI at post treatment yielded no significant difference in variance ratios (level 1: lnVR = −0.02; 95% credible interval [CrI], −0.07 to 0.03). Level 2 (lnVR = 0.09; 95% CrI, 0.05 to 0.13) and level 3 estimates (lnVR = 0.07; 95% CrI, 0.01 to 0.12) confirmed the 3-level analysis model.

The only significant variable associated with HTE was baseline severity (β̂ = 0.04; 95% CrI, 0.01 to 0.07), indicating higher HTE in populations with greater depression severity. All other sensitivity and subgroup analyses showed a near-constant ES throughout the investigated study design, intervention, and participant characteristics (Table). Extended results can be found in eAppendix 8 in Supplement 1.

**Table.** Bayesian 3-Level Meta-Regression Results for HTE in Internet- and Mobile-Based Interventions for Depression^a^

Outcome	Studies, No.	Level 1 (95% CrI)	Level 2 (95% CrI)	Level 3 (95% CrI)
Primary outcome				
Posttreatment HTE	102	−0.02 (−0.07 to 0.03)	0.09 (0.05 to 0.13)	0.07 (0.01 to 0.12)
Subgroups analysis				
Study design characteristics				
Setting				
Effectiveness	24	−0.05 (−0.14 to 0.04)	0.04 (0.00 to 0.11)	0.10 (0.02 to 0.16)
Efficacy	78	0.00 (−0.07 to 0.07)	0.10 (0.06 to 0.14)	0.07 (0.01 to 0.13)
Control type				
Waiting list control	47	0.06 (−0.06 to 0.16)	0.13 (0.09 to 0.18)	0.06 (0.00 to 0.13)
TAU	27	−0.02 (−0.11 to 0.07)	0.05 (0.00 to 0.12)	0.09 (0.01 to 0.16)
Attention control	24	−0.05 (−0.14 to 0.04)	0.03 (0.00 to 0.10)	0.07 (0.01 to 0.14)
Intervention characteristics				
Guidance				
Guided	56	0.02 (−0.07 to 0.11)	0.10 (0.04 to 0.15)	0.08 (0.01 to 0.14)
Technical guidance	27	−0.07 (−0.16 to 0.02)	0.09 (0.02 to 0.15)	0.06 (0.00 to 0.13)
Unguided	25	−0.01 (−0.12 to 0.10)	0.05 (0.00 to 0.14)	0.11 (0.01 to 0.19)
Therapeutic background				
CBT	71	−0.01 (−0.08 to 0.05)	0.05 (0.00 to 0.10)	0.10 (0.03 to 0.14)
Third wave	14	−0.02 (−0.23 to 0.19)	0.06 (0.00 to 0.18)	0.10 (0.01 to 0.23)
Technology				
Internet-based	88	−0.03 (−0.08 to 0.03)	0.09 (0.05 to 0.13)	0.07 (0.01 to 0.12)
Other				
Publication period				
Last decade (≥2013)	82	−0.01 (−0.07 to 0.04)	0.10 (0.06 to 0.14)	0.07 (0.01 to 0.13)
Last 5 y (≥2018)	39	−0.03 (−0.13 to 0.06)	0.14 (0.09 to 0.19)	0.07 (0.00 to 0.16)
Post assessment time				
<6 mo	102	−0.00 (−0.05 to 0.04)	0.08 (0.06 to 0.11)	0.09 (0.05 to 0.12)
6 to 12 mo	21	−0.00 (−0.10 to 0.10)	0.03 (0.00 to 0.07)	0.03 (0.00 to 0.08)
≥1 y	16	−0.02 (−0.16 to 0.14)	0.03 (0.00 to 0.09)	0.06 (0.00 to 0.13
Moderation analysis				
Participant characteristics				
Age	94	0.01 (−0.02 to 0.04)	0.10 (0.06 to 0.14)	0.07 (0.01 to 0.12)
Percentage female	98	0.02 (−0.01 to 0.04)	0.09 (0.04 to 0.13)	0.07 (0.01 to 0.12)
Baseline severity	99	0.04 (0.01 to 0.07)	0.09 (0.04 to 0.12)	0.07 (0.01 to 0.11)
Other				
Assessment time		NA	0.08 (0.05 to 0.10)	0.08 (0.05 to 0.11)
Linear	102	−0.00 (−0.01 to 0.01)	NA	NA
Quadratic	102	−0.00 (−0.00 to 0.00)	NA	NA

Abbreviations: CBT, cognitive behavioral therapy; CrI, credible interval; HTE, heterogeneity of treatment effects; NA, not applicable; TAU, treatment as usual.

^a^ Level 1 estimates in primary outcome and subgroup analysis quantify the HTE (logarithmic variance ratio μ̂; zero indicates equivalent variances in the intervention and control groups). Moderation analysis presents the association of the investigated variables with HTE (β̂ zero indicates no effect on HTE). Level 2 estimates quantify how much estimates vary within studies. Level 3 estimates quantify the extent to which estimates vary between studies. 95% CrIs quantify the 95% interval in which the true estimate lies given the provided data. For extended results, including adjusting covariate estimate (lnER), see eAppendix 8 in Supplement 1.

### Secondary Outcomes on Efficacy and Effectiveness

Secondary analyses of the posttreatment ESs of IMI for depression showed a medium ES favoring IMI (*g* = −0.56; 95% CrI, −0.46 to −0.66). While the ES for human-guided IMI (*g* = −0.62, 95% CrI, −0.50 to −0.75) compared with unguided IMI (*g* = −0.57, 95% CrI, −0.24 to −0.91) was higher, the difference was not significant overall (difference in *g*s, 0.07, 95% CrI, −0.17 to 0.31), in efficacy studies (difference in *g*s, 0.09, 95% CrI, −0.23 to 0.41), or in effectiveness studies (difference in *g*s, 0.01, 95% CrI, −0.25 to 0.27). From participant characteristics, only baseline severity was associated with the ES (β = −0.26, 95% CrI, −0.17 to −0.36). A significant interaction between guidance and baseline severity was found, suggesting the increased impact of therapeutic guidance with increasing baseline severity (interaction effect: β = −0.24, 95% CrI, −0.03 to −0.46). Detailed results on ES analyses can be found in eAppendix 9 in Supplement 1.

## Discussion

To our knowledge, the present study is the first of its kind to systematically investigate the HTE in IMI for depression to evaluate to what extent patient-by-treatment interactions exist. There was a lack of evidence supporting meaningful variability in treatment effects across various settings and populations (lnVR = −0.02, 95% CrI, −0.07 to 0.03). However, meta-regression analysis revealed that baseline severity affected HTE (β̂ = 0.04; 95% CrI, 0.01 to 0.07). Our findings indicate that patients with more severe depression responded differently to IMI and substantial patient-by-treatment interaction could be present in this subgroup. This supports that the average medium to large ES of IMI (*g* = −0.56; 95% CrI, −0.46 to −0.66) can be reasonably assumed for individuals with subthreshold to mild depression. In contrast, precision digital mental health care and future research are indicated for patients with moderate to severe depression to understand and counteract the increased HTE in that population.

Contrary to these findings for IMI, face-to-face psychotherapeutic approaches for depression show an overall HTE (lnVR = 0.09; 95% CrI, 0.06 to 0.14).^27^ A key difference between IMI and face-to-face psychotherapy is the extent to which treatment protocols can be standardized and enforced.^145,146^ While this might be a potential explanation for the difference in findings in face-to-face psychotherapy and the similarity to findings for other standardized treatment options (eg, antidepressants),^23,27^ it is important to note that the present study design does not allow for causal interpretations. The finding regarding baseline severity and HTE does not mean that HTE is caused by the severity itself. Rather, it calls for future studies to investigate the causes of HTE in patients with mild to severe depression to pave the way toward precision digital depression treatment, where IMIs are only recommended to those patients with higher depression severity who are likely to benefit from IMI. Studies at the individual level, such as individual-patient data meta-analyses, could be promising to comprehend these diverse response patterns and their underlying causes.^147^ Besides, our understanding of the mechanism of IMI is still limited, which makes it hard to explain why IMIs work.^31,148,149^ More in-depth studies on the underlying mechanisms of change and effect analysis of specific components are highly needed to understand and optimize IMI for the treatment of depression.^31,148,149,150,151^

Regarding components of IMI, we found that human therapeutic guidance was associated with increased treatment effects from IMI in populations with higher depression severity (interaction effect: β̂ = −0.24; 95% CrI, −0.03 to −0.46). Extending previous findings by Karyotaki and colleagues,^12^ findings indicated no general superiority of guided interventions compared with unguided. Guidance did not provide an incremental benefit for subthreshold to mild depression; this difference was only found in higher levels of depression. This distinction may also explain the so-far inconsistent findings regarding the benefits of guidance.^11,12,31,32^ Importantly, we did not find evidence for substantial HTE in the subset of guided IMIs, despite the involvement of human therapeutic elements (eg, personalized feedback and recommendations), which remove standardization to some extent. This option to personalize feedback and content to the patient may be a central reason why guidance was associated with the outcomes of IMI in higher severity.

For clinical practice, the key finding is that we replicated previous findings on the moderate to large ESs of IMIs throughout the efficacy and effectiveness analyses with an unprecedented sample size of nearly 20 000 participants.^11,12^ Especially the increase in effectiveness studies in recent years and the meta-analytically small to medium ES (*g* = −0.30; 95% CrI, −0.16 to −0.43) in studies conducted in pragmatic clinical settings^11^ highlights the clinical value of IMIs beyond the laboratory. In conjunction with the results on the HTE and the associations of baseline severity and guidance, our findings suggest that unguided IMI can serve as standardized treatment for subthreshold to mild depression, showing clinically relevant meta-analytical effectiveness and no evidence for increased patient-by-treatment interactions (ie, clinically relevant mean differences but equivalent variances to active and passive control groups). In contrast, evidence for increased patient-by-treatment in moderate to severe depression indicates the need for a personalized precision approach for this population, for whom IMIs should only be used in yet-to-be-identified responding subgroups and human therapeutic guidance is involved (eg, to be able to react to the specific needs of individual patients). Similar to guidance, other dimensions to personalize IMIs to the individual (eg, content, order, or communication) might be able to reduce the HTE in this subgroup.^152^ However, future RCTs investigating the effectiveness of these approaches are needed before robust recommendations can be made for patients with severe depression.

### Limitations

When interpreting the present results, some limitations should be considered. First, we found within-study (level 2) and between-study (level 3) variances indicating differences between studies. To take these into account we used a 3-level bayesian meta-regression model and conducted sensitivity and subgroup analyses in more homogeneous studies, which replicated the core findings. However, level 2 and level 3 variances remained, and given the limited details provided in the included studies, we cannot rule out that operationalization of control types, implementation (eg, the extent or uptake of guidance), and settings differed between studies. Future studies should aim to reduce these variances by analyzing additional factors, such as differences between intervention components (eg, therapeutic content) or intervention design features (eg, persuasive design, engagement, aesthetics).^11,31,153,154,155^

Second, an aspect of particular importance for future studies is the type of technology used in IMIs. Most included studies were focused on internet-based interventions, allowing strong conclusions for this method of treatment delivery. However, other formats, such as smartphone- and app-based interventions, exist and are currently rising in the market.^11,14,154,156,157,158,159^ The current body of evidence on the efficacy of mobile applications for depression is limited by the scarcity of high-quality RCTs, particularly in pragmatic clinical settings that use active control groups.^14,154,158,160^ Moreover, due to the limited number of studies, reliable subgroup analyses were not feasible in this meta-analysis. As a result, caution should be exercised in generalizing the present findings of mainly internet-based interventions to app-based interventions or digital therapeutics in general.^11,14,150,154,157,158,161^

Third, given the limited sample variety, the generalizability of the present findings must be questioned. Except for 8 studies conducted in Asia, the vast majority of trials were conducted in Western industrialized countries, limiting the generalizability to other cultures and backgrounds.^162^ Additionally, with roughly 70% of the participants being female, male participants and those with other genders are underrepresented in the current literature. As this is the first study of which we are aware investigating the HTE in IMIs, replications are needed to determine the generalizability of the findings, which is particularly true for these so-far-underrepresented samples in the field.

Additionally, although essential for evidence-based medicine and recommendations in treatment guidelines, meta-analyses on properly conducted RCTs remain exploratory. Hence, in particular, the here-found moderation results cannot replace the need for adequately designed confirmatory studies validating the findings of this study (eg, the superiority of guided IMI over unguided IMI in populations with higher severity levels). Furthermore, the present meta-analysis focused on depression severity and does not allow for generalization to other important outcomes, such as reliable improvement and deterioration, which require separate analysis in future studies.

## Conclusions

In this systematic review and meta-analysis of the HTE of IMIs for depression, the equivalence in variance in IG and CG suggested that the average moderate treatment effect of IMI can be reasonably assumed for individuals with subthreshold to mild depression. However, HTE increased in individuals with moderate to high depression severity, indicating patient-by-treatment interaction and subgroups in this population particularly nonresponding. Future research on the causes of individual responses in this target group is required. Based on the current evidence, the use of unguided IMI for subthreshold to mild depression can be strongly recommended for clinical practice and policy as an effective, low-resource, time- and location-independent treatment. However, more studies are needed before the recommendation of IMIs can be generalized to populations with higher symptom burdens. Furthermore, human therapeutic guidance should be a central component moving forward to the treatment of individuals with more severe symptoms using IMIs.

## Notes

Supplement 1. **eAppendix 1.** Illustration of Mean and Variance Differences in an RCT **eAppendix 2.** Search String **eMethods 1.** Model Parameters and Interpretation **eMethods 2.** Priors **eMethods 3**. HTE Subgroup and Sensitivity Analysis **eMethods 4.** Effects Meta-Analysis **eAppendix 3.** R Packages **eAppendix 4.** Search Results and Flow Chart **eAppendix 5.** Dataset **eAppendix 6.** Individual Risk of Bias Ratings **eAppendix 7.** Risk of Bias and Study Quality: Sensitivity Analysis **eAppendix 8.** Extended Results **eAppendix 9.** Efficacy and Effectiveness Analysis Results

Supplement 2. **eTable.** Study Characteristics

Supplement 3.
Data Sharing Statement
