# Supplementary material for: Heterogeneity of Treatment Effects in Internet- and Mobile-Based Interventions for Depression: A Systematic Review and Meta-Analysis
Source: JAMA Netw Open. 2024 Jul 18;7(7):e2423241. doi: 10.1001/jamanetworkopen.2024.23241 (PMC11258589; doi:10.1001/jamanetworkopen.2024.23241)
Supplement: Supplement 1. — eAppendix 1. Illustration of Mean and Variance Differences in an RCT eAppendix 2. Search String eMethods 1. Model Parameters and Interpretation eMethods 2. Priors eMethods 3. HTE Subgroup and Sensitivity Analysis eMethods 4. Effects Meta-Analysis eAppendix 3. R Packages eAppendix 4. Search Results and Flow Chart eAppendix 5. Dataset eAppendix 6. Individual Risk of Bias Ratings eAppendix 7. Risk of Bias and Study Quality: Sensitivity Analysis eAppendix 8. Extended Results eAppendix 9. Efficacy and Effectiveness Analysis Results [file jamanetwopen-e2423241-s001.pdf]

Supplemental Online Content

Terhorst Y, Kaiser T, Brakemeier E, et al. Heterogeneity of treatment effects in internet- and mobile-based interventions for depression. *JAMA Netw Open.* 2024;7(7):e2423241. doi:10.1001/jamanetworkopen.2024.23241

eAppendix 1. Illustration of Mean and Variance Differences in an RCT

eAppendix 2. Search String

eMethods 1. Model Parameters and Interpretation

eMethods 2. Priors

eMethods 3. HTE Subgroup and Sensitivity Analysis

eMethods 4. Effects Meta-Analysis

eAppendix 3. R Packages

eAppendix 4. Search Results and Flow Chart

eAppendix 5. Dataset

eAppendix 6. Individual Risk of Bias Ratings

eAppendix 7. Risk of Bias and Study Quality: Sensitivity Analysis

eAppendix 8. Extended Results

eAppendix 9. Efficacy and Effectiveness Analysis Results

This supplemental material has been provided by the authors to give readers additional information about their work.

© 2024 Terhorst Y et al. *JAMA Network Open.*

eAppendix 1. Illustration of Mean and Variance Differences in an RCT

Assuming a randomized controlled trial with one intervention group (IG) and one control group (CG), the mean (e.g., an average of depression severity) and standard deviation (e.g., the sum of the individual differences from the mean) are two important points of interests when evaluating the effects of an intervention. By allocating patients randomly to either the IG or the CG, patient characteristics are distributed randomly between the groups, including variables potentially influencing the outcome. Hence, any difference in the outcome can be only explained by the intervention. Following this rationale, a typical observation made after randomization is the finding of equal means and standard deviations in both groups (see eAppendix 1, Figure 1).

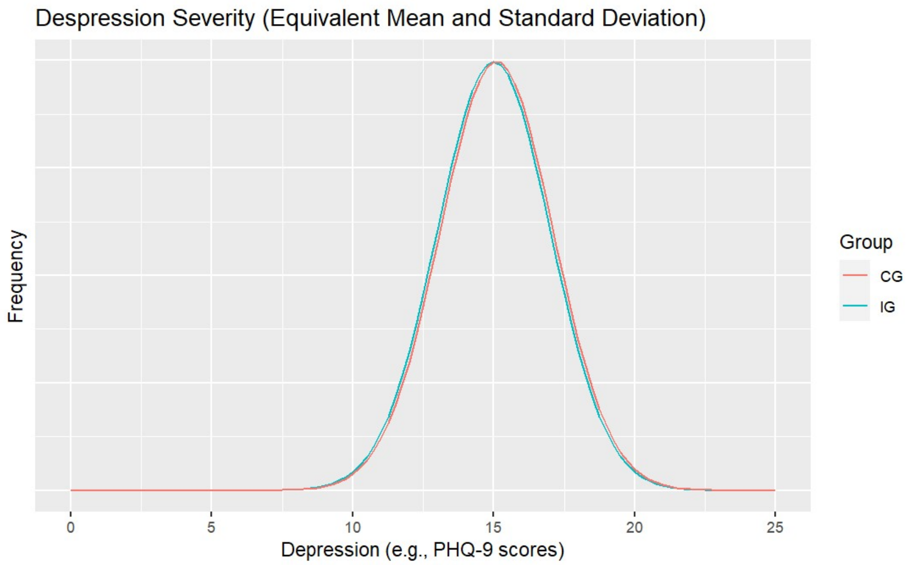

eAppendix1, Figure 1. Illustration at Baseline

If the intervention is effective a mean difference is present at post-treatment assessment. However, an important point is that not all treated patients have the same severity value after treatment. In contrast, a variance of post-treatment depression severity is observable both in the IG and the CG (see eAppendix 1, Figure 2). It is important to note that in this example, the variances are equivalent between the IG and CG. This indicates that the intervention is effective (i.e., lower observable mean depression severity) but that the extent of individual responses (i.e., variance) is equivalent in the IG and CG. Hence, variance ratios would be close to one or zero in case the logarithm is used.

© 2024 Terhorst Y et al. *JAMA Network Open.*

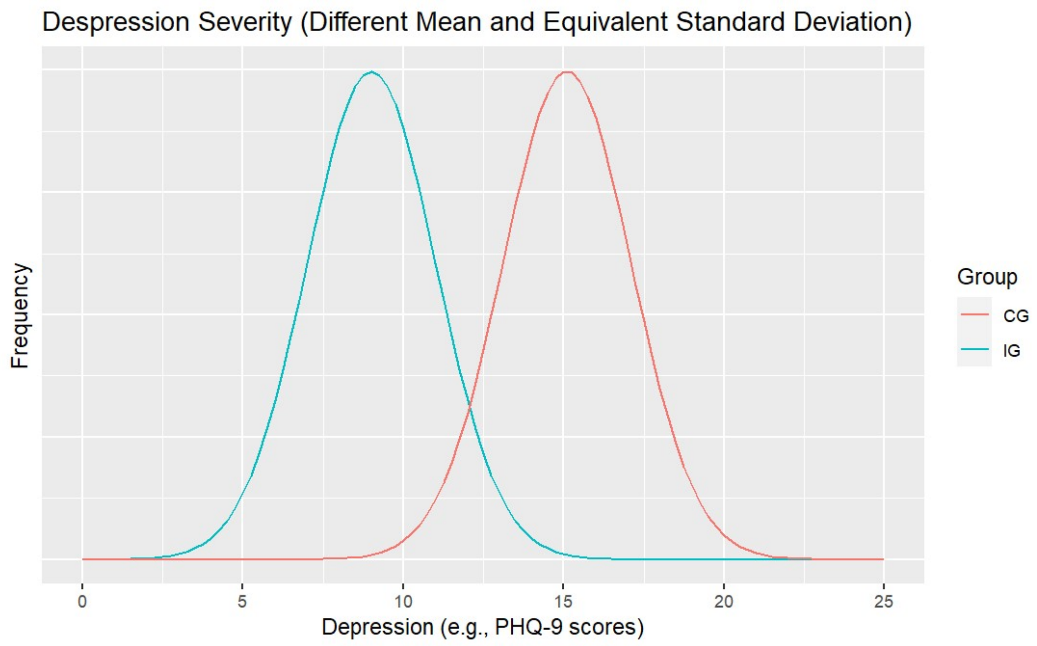

eAppendix1, Figure 2. Illustration of Mean Differences and Equivalent Variances

In contrast to the finding illustrated in eAppendix1 Figure 2, a different result in an RCT could be that unequal mean depression severity is observed as well as unequal variances in the IG and CG (eAppendix 1, Figure 3).

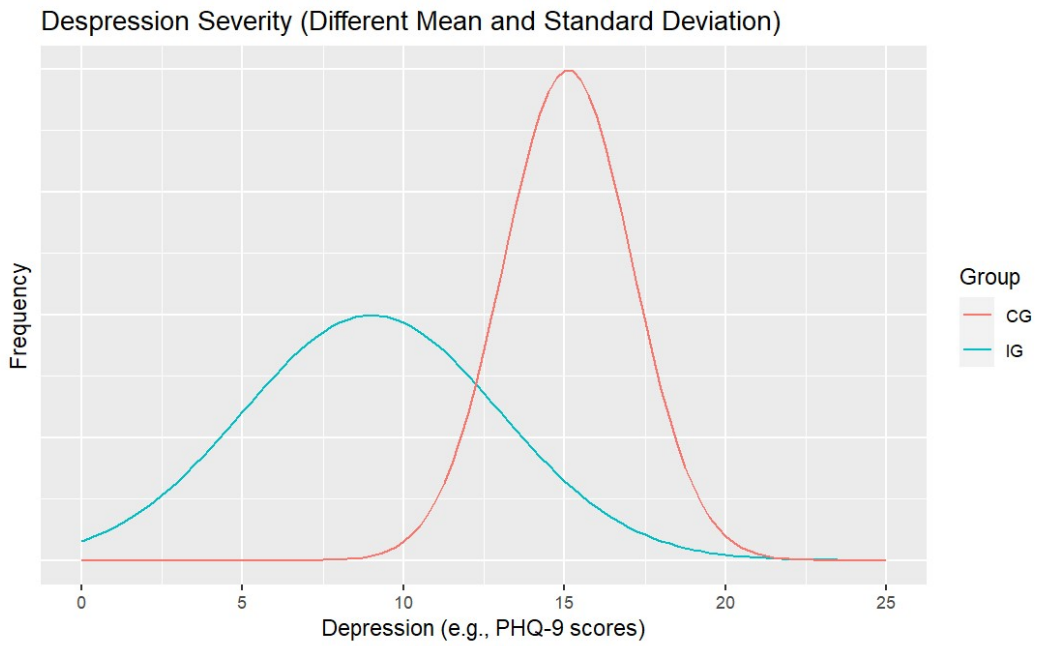

eAppendix1, Figure 3. Illustration of Mean Differences and Differences in Variances

© 2024 Terhorst Y et al. *JAMA Network Open.*

This again would indicate that the intervention is effective and causes a lower observable mean severity in the IG compared to the CG. However, simultaneously, this shows that a much stronger variance in depression severity is observable in the IG compared to the CG (i.e., higher variation in response to treatment indicating treatment-by-patient interactions). As for the mean differences, this unequal ratio of variance can be interpreted as a causal result from the intervention due to the randomization, which suggests that the intervention is particularly (in)effective in some patients.

Similarly to the mean differences, a meta-analysis can systematically investigate the variance ratios. If the variance ratios between IG and CG across RCTs are systematically different, this would provide evidence that the extent of individual responses is stronger in one of the groups. As a result, this would question whether a meta-analytically average effect can be reasonably assumed for the individual and instead indicates the presence of substantial treatment-by-patient interactions. Furthermore, a meta-analysis could conduct subgroup and meta-regression analysis to provide first insights into the subgroups of patients where treatment-by-patient interactions exist. For instance, unequal variances might be observable in a subgroup of RCTs evaluating a specific type of intervention (e.g., face-to-face psychotherapy or guided Internet- and mobile-based interventions) but equivalent in another subgroup of interventions (e.g., antidepressants or unguided Internet- and mobile-based interventions). However, a very important point in interpreting equivalent variance ratios in meta-analysis is that it does not mean there is no variance in treatment effect. In contrast, it provides insights into whether the variances are in the same range “normally” observed in other treatments (e.g., if compared to active control groups) or no treatment (e.g., if compared to wait-list control groups).

Please note this is a conceptual introduction to the here applied methodology, simplifying various important points for improved comprehensibility (e.g., the relationship between mean and variance or the characteristics of variance ratios compared to the logarithm of variance ratios). For a more in-depth introduction, we recommend the following articles:

Nakagawa S, Poulin R, Mengersen K, et al. Meta-analysis of variation: ecological and evolutionary applications and beyond. *Methods Ecol Evol*. 2015;6(2):143-152. doi:10.1111/2041-210X.12309

Volkmann C, Volkmann A, Müller CA. On the treatment effect heterogeneity of antidepressants in major depression: A Bayesian meta-analysis and simulation study. Hutson AD, ed. *PLoS One*. 2020;15(11):e0241497. doi:10.1371/journal.pone.0241497

Winkelbeiner S, Leucht S, Kane JM, Homan P. Evaluation of Differences in Individual Treatment Response in Schizophrenia Spectrum Disorders. *JAMA Psychiatry*. 2019;76(10):1063. doi:10.1001/jamapsychiatry.2019.1530

Importantly, the concept of heterogeneity of treatment effects differs fundamentally from the statistical between-study heterogeneity often reported in meta-analysis. The latter refers to the extent the true effectsize varies in a meta-analysis. Similarly to the between-study heterogeneity of the effect size of mean differences (e.g., Hedges' g) in a meta-analysis, also other effect sizes like the variance ratio can show between-study heterogeneity (i.e., the extent of the true variance ratio varies within the meta-analysis). For a more detailed explanation of between-study heterogeneity in meta-analysis see Harrer and colleagues (2021).

Harrer, M., Cuijpers, P., Furukawa, T., & Ebert, D. (2021). *Doing meta-analysis with R: A hands-on guide*. Chapman and Hall/CRC.

eAppendix 2. Search String

SEARCH #	MEDLINE	CENTRAL	PSYCINFO	EMBASE
1	exp Internet/	exp Internet/	exp Internet/	internet'/exp OR internet
2	exp Cell Phone/	exp Cell Phone/	exp Mobile Devices/	mobile phone'/exp
3	exp Mobile Applications/	exp Mobile Applications/	exp Computer Applications/	mobile application'/exp
4	internet*.mp.	internet*.mp.	internet*.mp.	internet*:ab,ti
5	online*.mp.	online*.mp.	online*.mp.	online*:ab,ti
6	web*.mp.	web*.mp.	web*.mp.	web*:ab,ti
7	computer*.mp.	computer*.mp.	computer*.mp.	computer*:ab,ti
8	mobile*.mp.	mobile*.mp.	mobile*.mp.	mobile*:ab,ti
9	1 OR 2 OR 3 OR 4 OR 5 OR 6 OR 7 OR 8	1 OR 2 OR 3 OR 4 OR 5 OR 6 OR 7 OR 8	1 OR 2 OR 3 OR 4 OR 5 OR 6 OR 7 OR 8	1 OR 2 OR 3 OR 4 OR 5 OR 6 OR 7 OR 8
10	exp Psychotherapy/	exp Psychotherapy/	exp Psychotherapy/	psychotherapy'/exp
11	psychotherap*.mp.	psychotherap*.mp.	psychotherap*.mp.	psychotherap*:ab,ti
12	"cognitive therap*".mp.	"cognitive therap*".mp.	"cognitive therap*".mp.	cognitive therap*':ab,ti
13	"cognitive behav*".mp.	"cognitive behav*".mp.	"cognitive behav*".mp.	cognitive behav*':ab,ti
14	10 OR 11 OR 12 OR 13	10 OR 11 OR 12 OR 13	10 OR 11 OR 12 OR 13	10 OR 11 OR 12 OR 13
15	9 AND 14	9 AND 14	9 AND 14	9 AND 14
16	exp Telemedicine/	exp Telemedicine/	exp Telemedicine/	telemedicine'/exp
17	exp Therapy, Computer-Assisted/	exp Therapy, Computer-Assisted/	exp Computer-Assisted Therapy/	computer assisted therapy'/exp
18	exp Distance Counseling/	"Distance Counseling".mp.	exp Online Therapy/	teletherapy'/exp
19				e counseling'/exp
20	E-health*.mp.	E-health*.mp.	E-health*.mp.	e health*':ab,ti
21	ehealth*.mp.	ehealth*.mp.	ehealth*.mp.	ehealth*:ab,ti
22	"telemedicine*".mp.	"telemedicine*".mp.	"telemedicine*".mp.	telemedicine*':ab,ti
23	"tele-medicine*".mp.	"tele-medicine*".mp.	"tele-medicine*".mp.	tele-medicine*':ab,ti
24	"telehealth*".mp.	"telehealth*".mp.	"telehealth*".mp.	telehealth*':ab,ti
25	"tele-health*".mp.	"tele-health*".mp.	"tele-health*".mp.	tele-health*':ab,ti
26	"tele-based*".mp.	"tele-based*".mp.	"tele-based*".mp.	tele-based*':ab,ti
27	Internet-based.mp.	Internet-based.mp.	Internet-based.mp.	internet based':ab,ti
28	"e-therap*".mp.	"e-therap*".mp.	"e-therap*".mp.	e-therap*':ab,ti
29	"e-mental health".mp.	"e-mental health".mp.	"e-mental health".mp.	e-mental health':ab,ti
30	emental health.mp.	emental health.mp.	emental health.mp.	emental health':ab,ti
31	telecare.mp.	telecare.mp.	telecare.mp.	telecare:ab,ti
32	iCBT.mp.	iCBT.mp.	iCBT.mp.	icbt:ab,ti
33	i-CBT.mp.	i-CBT.mp.	i-CBT.mp.	i cbt':ab,ti
34	cCBT.mp.	cCBT.mp.	cCBT.mp.	ccbt:ab,ti
35	c-CBT.mp.	c-CBT.mp.	c-CBT.mp.	c cbt':ab,ti
36	16 OR 17 OR 18 OR 20 OR 21 OR 22 OR 23 OR 24 OR 25 OR 26 OR 27 OR 28 OR 29 OR 30 OR 31 OR 32 OR 33 OR 34 OR 35	16 OR 17 OR 18 OR 20 OR 21 OR 22 OR 23 OR 24 OR 25 OR 26 OR 27 OR 28 OR 29 OR 30 OR 31 OR 32 OR 33 OR 34 OR 35	16 OR 17 OR 18 OR 20 OR 21 OR 22 OR 23 OR 24 OR 25 OR 26 OR 27 OR 28 OR 29 OR 30 OR 31 OR 32 OR 33 OR 34 OR 35	16 OR 17 OR 18 OR 19 OR 20 OR 21 OR 22 OR 23 OR 24 OR 25 OR 26 OR 27 OR 28 OR 29 OR 30 OR 31 OR 32 OR 33 OR 34 OR 35
37	15 OR 36	15 OR 36	15 OR 36	15 OR 36
38	exp Mood Disorders/	exp Mood Disorders/	exp Mood Disorders/	mood disorder'/exp
39	exp Depression/	exp Depression/	exp Depression/	depression'/exp
40	exp Depressive Disorder/	exp Depressive Disorder/		
41	"depressi*".mp.	"depressi*".mp.	"depressi*".mp.	depressi*':ab,ti
42	"mood disorder*".mp.	"mood disorder*".mp.	"mood disorder*".mp.	mood disorder*':ab,ti
43	"affective disorder*".mp.	"affective disorder*".mp.	"affective disorder*".mp.	affective disorder*':ab,ti
44	"dysthymi*".mp.	"dysthymi*".mp.	"dysthymi*".mp.	dysthymi*':ab,ti
45	38 OR 39 OR 40 OR 41 OR 42 OR 43 OR 44	38 OR 39 OR 40 OR 41 OR 42 OR 43 OR 44	38 OR 39 OR 41 OR 42 OR 43 OR 44	38 OR 39 OR 41 OR 42 OR 43 OR 44
46	37 AND 45	37 AND 45	37 AND 45	37 AND 45
47	exp Randomized Controlled Trials as Topic/	exp Randomized Controlled Trials as Topic/		randomized controlled trial (topic)'/exp
48	randomi?ed.ti,ab.	randomi?ed.ti,ab.	randomi?ed.ti,ab.	randomi?ed:ab,ti
49	placebo.ti,ab.	placebo.ti,ab.	placebo.ti,ab.	placebo:ab,ti
50	randomly.ti,ab.	randomly.ti,ab.	randomly.ti,ab.	randomly:ab,ti
51	trial.ti,ab.	trial.ti,ab.	trial.ti,ab.	trial:ab,ti
52	groups.ti,ab.	groups.ti,ab.	groups.ti,ab.	groups:ab,ti
53	controlled clinical trial.mp.	controlled clinical trial.mp.	controlled clinical trial.mp.	controlled clinical trial':ab,ti
54	random*.mp.	random*.mp.	random*.mp.	random*:ab,ti
55	"randomi?ed controlled trial".mp.	"randomi?ed controlled trial".mp.	"randomi?ed controlled trial".mp.	randomi?ed controlled trial':ab,ti
56	"controlled clinical trial".mp.	"controlled clinical trial".mp.	"controlled clinical trial".mp.	controlled clinical trial':ab,ti
57	controll*.mp.	controll*.mp.	controll*.mp.	controll*:ab,ti
58	RCT.mp.	RCT.mp.	RCT.mp.	rct:ab,ti
59	47 OR 48 OR 49 OR 50 OR 51 OR 52 OR 53 OR 54 OR 55 OR 56 OR 57 OR 58	47 OR 48 OR 49 OR 50 OR 51 OR 52 OR 53 OR 54 OR 55 OR 56 OR 57 OR 58	48 OR 49 OR 50 OR 51 OR 52 OR 53 OR 54 OR 55 OR 56 OR 57 OR 58	47 OR 48 OR 49 OR 50 OR 51 OR 52 OR 53 OR 54 OR 55 OR 56 OR 57 OR 58
60	46 AND 59	46 AND 59	46 AND 59	46 AND 59

© 2024 Terhorst Y et al. *JAMA Network Open.*

eMethods 1. Model Parameters and Interpretation

Throughout the analyses we applied a three-level Bayesian meta-regression model. The key parameters of interest in the three-level regression models are 1) the intercept ($\hat{\mu}$), which indicates whether the ratio of variance between IG and CG significantly differs from zero and, hence, substantial patient-by-treatment interactions exist, and 2) the estimate of moderators ($\hat{\beta}$) on the estimated lnVR, which indicates whether the influence of the moderator is significant. We consistently employed 95% credibility intervals (CrI) in all analyses to gauge uncertainty. The 95%-CrI of the posterior distribution of a parameter provides an interval wherein the true parameter lies with a 95% probability. If the 95%-CrI does include zero for the HTE effect size (lnVR), the assumption of different variances in IG and CG would be implausible. In addition, we accounted for between-observation variance (level II) and between-study variance (level III) of the true effect size. If 95%-CrI of level II and level III estimates include zero, the modeling of these estimates would be unnecessary, implying a simpler model structure. For an in-depth introduction to three-level meta-regression, please refer to:

Assink M, Wibbelink CJM. Fitting three-level meta-analytic models in R: A step-by-step tutorial. 2016;12(3):154-174.

Bürkner PC. {brms}: An {R} Package for {Bayesian} Multilevel Models Using {Stan}. J Stat Softw. 2017;80(1):1-28. doi:10.18637/jss.v080.i01

Volkmann C, Volkmann A, Müller CA. On the treatment effect heterogeneity of antidepressants in major depression: A Bayesian meta-analysis and simulation study. Hutson AD, ed. PLoS One. 2020;15(11):e0241497. doi:10.1371/journal.pone.0241497

eMethods 2. Priors

We selected weak priors in all analyses:

$$\mu \sim Cauchy(0, 1)$$

$$\beta \sim Cauchy(0, 1)$$

$$\tau \sim Cauchy(0, 0.5)$$

Note: μ (=intercept estimate), β (=predictor/moderator estimate), τ variance between outcomes within a study (level 2) and variance between studies (level 3).

To explore the impact of priors we additionally ran analyses using the posterior estimates obtained in HTE analyses of face-to-face psychotherapy from Kaiser et al. yielding no meaningful differences.

Kaiser T, Volkmann C, Volkmann A, Karyotaki E, Cuijpers P, Brakemeier EL. Heterogeneity of treatment effects in trials on psychotherapy of depression. *Clin Psychol Sci Pract*. 2022;29(3):294-303. doi:10.1037/cps0000079

© 2024 Terhorst Y et al. *JAMA Network Open.*

eMethods 3. HTE Subgroup and Sensitivity Analysis

To investigate the role of various design and study characteristics, intervention characteristics, participant characteristics, and potential long-term HTE, we conducted subgroup and sensitivity analyses as follows:

Design and study characteristics: The potential influence of the study setting (efficacy studies and effectiveness studies) was investigated in respective subgroup analyses (e.g., a subset of only effectiveness studies). As defined in previous studies[3–5], we coded studies as efficacy studies if they investigated effects in highly controlled circumstances (i.e., resource-intensive setting, restrictive in- and exclusion criteria, unrepresentative highly trained personnel, and standardized, strictly enforced intervention). In contrast, an effectiveness study is characterized by a real-world setting, a heterogeneous sample resulting from few to no exclusion criteria, personnel representative of usual care providers, and access to treatment as usual and other parallel interventions. In addition to the study setting, we investigated the type of control group by separately analysing RCTs following a waitlist control group, attention control, and treatment-as-usual design.

Intervention characteristics: IMI vary highly in the extent of human-provided support. Hence, we conducted subgroup analyses for RCTs including a) therapeutically guided interventions (therapeutic support by a human), b) technically guided interventions (compliance-focused support by a human), and c) unguided interventions (no support by a human). Furthermore, we investigated the theoretical background of IMI (i.e., cognitive behavioural therapy, or third-wave therapy) and the technology type (e.g., Internet-based, smartphone app based). Lastly, due to the fast developments in technology, additional subgroup analyses were conducted limited to studies published in the last decade (2013 to 2023) and five years (2018 to 2023).

Participant characteristics: For participant characteristics, we included the average age of participants in a study and the percentage of female participants as a covariate in the in the meta-regression model. In addition, we investigated the baseline severity expressed in PHQ-9 (*z*-standardized) as a predictor of HTE. Recoding of depression instruments into PHQ-9 was performed

© 2024 Terhorst Y et al. *JAMA Network Open.*

by two independent researchers (YT and PP) using standardized conversion equations[6] and common-metric cross-walk tables.[4,7,8]

Long-term HTE: Lastly, we shifted the analysis away from only post-treatment assessments to all assessments (i.e., including all assessment points of a study). Assessment time (i.e., time after baseline assessment) was included as a covariate to investigate HTE over time as a linear and quadratic predictor. Moreover, we conducted subgroup analyses in subsets only including comparisons between IG and CG within a) the first six months, b) six to twelve months, and c) over one year after the baseline assessment.

1. Assink M, Wibbelink CJM. Fitting three-level meta-analytic models in R: A step-by-step tutorial. 2016;12(3):154-174.
2. Bürkner PC. {brms}: An {R} Package for {Bayesian} Multilevel Models Using {Stan}. *J Stat Softw*. 2017;80(1):1-28. doi:10.18637/jss.v080.i01
3. Moshe I, Terhorst Y, Cuijpers P, Cristea I, Pulkki-Råback L, Sander L. Three Decades of Internet- and Computer-Based Interventions for the Treatment of Depression: Protocol for a Systematic Review and Meta-Analysis. *JMIR Res Protoc*. 2020;9(3):e14860. doi:10.2196/14860
4. Moshe I, Terhorst Y, Philippi P, et al. Digital interventions for the treatment of depression: A meta-analytic review. *Psychol Bull*. 2021;147(8):749-786. doi:10.1037/bul0000334
5. Singal AG, Higgins PDR, Waljee AK. A primer on effectiveness and efficacy trials. *Clin Transl Gastroenterol*. 2014;5(1):e45. doi:10.1038/ctg.2013.13
6. Hawley CJ, Gale TM, Smith PSJ, et al. Equations for converting scores between depression scales (MÅDRS, SRS, PHQ-9 and BDI-II): good statistical, but weak idiographic, validity. *Hum Psychopharmacol Clin Exp*. 2013;28(6):544-551. doi:10.1002/hup.2341
7. Wahl I, Löwe B, Bjorner JB, et al. Standardization of depression measurement: a common metric was developed for 11 self-report depression measures. *J Clin Epidemiol*. 2014;67(1):73-86. doi:10.1016/J.JCLINEPI.2013.04.019
8. Choi SW, Schalet B, Cook KF, Cella D. Establishing a common metric for depressive symptoms: Linking the BDI-II, CES-D, and PHQ-9 to PROMIS Depression. *Psychol Assess*. 2014;26(2):513-527. doi:10.1037/a0035768

© 2024 Terhorst Y et al. *JAMA Network Open.*

eMethods 4. Effects Meta-Analysis

In addition to the HTE analysis, we conducted a Bayesian three-level meta-regression on the effect size (ES) of IMI for depression. Therefore, we calculated Hedges' *g* between IG and CG (level I). Analogously to the HTE analysis, we handled studies with multiple outcomes by including all outcomes and estimating variances between outcomes within a study (level II) and variances between studies (level III). We applied ES analyses first to all post-treatment ES followed by sensitivity and subgroup analysis as outlined in the HTE analyses. Similarly, the key parameters (and 95%-CrI) of interest are 1) the intercept ($\hat{\mu}$), indicating whether the standardized mean difference (Hedges' *g)* between IG and CG differs from zero, and 2) the estimate of moderators ($\hat{\beta}$), indicating whether the estimated difference between IG and CG is influenced by the respective moderator. Sensitivity and subgroup analyses were conducted analogously to eMethods 1.

eAppendix 3. R Packages

Analysis script is open-accessible at https://osf.io/u3vdn/ under CC-By Attribution 4.0 international license.

For a quick overview of the used packages please see the session information below.

R version 4.2.2 (2022-10-31 ucrt)

Platform: x86_64-w64-mingw32/x64 (64-bit)

Running under: Windows 10 x64 (build 22621)

attached base packages:

stats graphics grDevices utils datasets methods base

other attached packages:

dmetar_0.0.9000 robvis_0.3.0 readxl_1.4.1 glue_1.6.2 ggridges_0.5.4 stringr_1.4.1 ggplot2_3.4.0 tidybayes_3.0.2 dplyr_1.0.10 tidyr_1.2.1 brms_2.18.0 Rcpp_1.0.9 loaded via a namespace (and not attached): metadat_1.2-0 backports_1.4.1 plyr_1.8.7 igraph_1.3.5 meta_6.0-0 splines_4.2.2 svUnit_1.0.6 crosstalk_1.2.0 rstantools_2.2.0 inline_0.3.19 digest_0.6.30 htmltools_0.5.3 fansi_1.0.3 magrittr_2.0.3 checkmate_2.1.0 cluster_2.1.4 RcppParallel_5.1.5 matrixStats_0.62.0 xts_0.12.2 prettyunits_1.1.1 colorspace_2.0-3 ggrepel_0.9.2 ggdist_3.2.0 xfun_0.34 callr_3.7.3 crayon_1.5.2 jsonlite_1.8.3 lme4_1.1-31 zoo_1.8-11 gtable_0.3.1 emmeans_1.8.2 V8_4.2.2 distributional_0.3.1 kernlab_0.9-31 pkgbuild_1.3.1 rstan_2.26.13 DEoptimR_1.0-11 prabclus_2.3-2 abind_1.4-5 scales_1.2.1 mvtnorm_1.1-3 DBI_1.1.3 miniUI_0.1.1.1 xtable_1.8-4 magic_1.6-1 mclust_6.0.0 stats4_4.2.2 StanHeaders_2.26.13 DT_0.26 netmeta_2.6-0 htmlwidgets_1.5.4 threejs_0.3.3 arrayhelpers_1.1-0 fpc_2.2-9 posterior_1.3.1 ellipsis_0.3.2 modeltools_0.2-23 pkgconfig_2.0.3 loo_2.5.1 flexmix_2.3-18 farver_2.1.1 nnet_7.3-18 utf8_1.2.2 tidyselect_1.2.0 rlang_1.0.6 reshape2_1.4.4 later_1.3.0 munsell_0.5.0 cellranger_1.1.0 tools_4.2.2 cli_3.3.0 generics_0.1.3 mathjaxr_1.6-0 evaluate_0.18 fastmap_1.1.0 yaml_2.3.6 processx_3.8.0 knitr_1.40 robustbase_0.95-0 purrr_0.3.5 nlme_3.1-160 mime_0.12 xml2_1.3.3 compiler_4.2.2 bayesplot_1.9.0 shinythemes_1.2.0 rstudioapi_0.14 curl_4.3.3 tibble_3.1.8 stringi_1.7.8 ps_1.7.2 Brobdingnag_1.2-9 lattice_0.20-

© 2024 Terhorst Y et al. *JAMA Network Open.*

45 poibin_1.5 Matrix_1.5-1 nloptr_2.0.3 markdown_1.3 shinyjs_2.1.0 tensorA_0.36.2 vctrs_0.5.0 CompQuadForm_1.4.3 pillar_1.8.1 lifecycle_1.0.3 bridgesampling_1.1-2 estimability_1.4.1 httpuv_1.6.6 R6_2.5.1 MuMIn_1.47.1 promises_1.2.0.1 gridExtra_2.3 codetools_0.2-18 boot_1.3-28 colourpicker_1.2.0 MASS_7.3-58.1 gtools_3.9.3 assertthat_0.2.1 withr_2.5.0 shinystan_2.6.0 metafor_3.8-1 diptest_0.76-0 parallel_4.2.2 grid_4.2.2 coda_0.19-4 class_7.3-20 minqa_1.2.5 rmarkdown_2.17 shiny_1.7.3 base64enc_0.1-3 dygraphs_1.1.1.6

© 2024 Terhorst Y et al. *JAMA Network Open.*

eAppendix 4. Search Results and Flow Chart

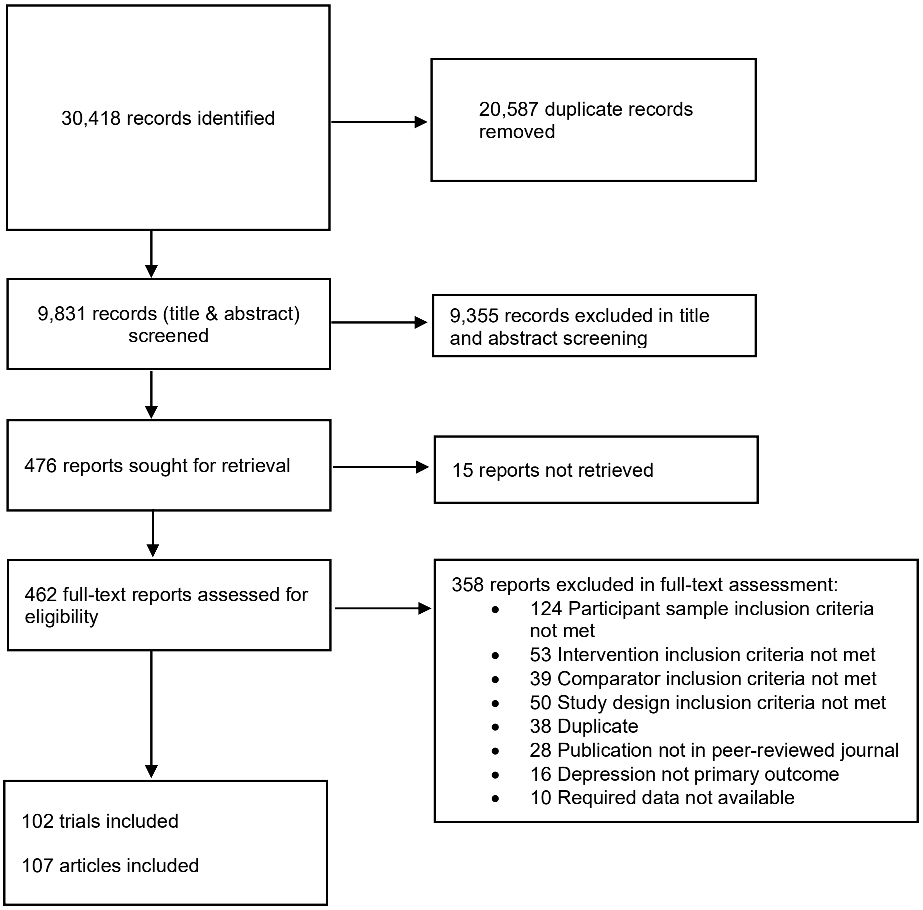

eFigure 1. PRISMA Flow-Chart

Note. Multiple studies can be based on the same trial (e.g., post-treatment effect sizes and follow-up effect sizes of a trial reported in separate articles). We refer to trials as original research projects, while articles is used for published research articles.

The updated search identified *k* = 19 new studies compared to Moshe and colleagues (2021), which represents an increase of 22.89% in the number of studies and *n* = 4228 (27.22%) in the number of involved patients. Due to this large number of included studies (*k* = 102), a digital filterable data set containing all the coded information, including participant, study design, and intervention characteristics, is provided online and open-accessible at https://osf.io/u3vdn/ (eAppendix7, supplement 1). See eAppendix 8 for a citation list of the included articles.

Moshe I, Terhorst Y, Philippi P, et al. Digital interventions for the treatment of depression: A meta-analytic review. *Psychol Bull*. 2021;147(8):749-786. doi:10.1037/bul0000334

eAppendix 5. Dataset

Due to the large number of studies, we provide a digital version of the data set with filter options to easily sort and identify the studies and data points included in the analysis respectively. Full data set, and analysis script is open-accessible at https://osf.io/u3vdn/ under CC-By Attribution 4.0 international license.

© 2024 Terhorst Y et al. *JAMA Network Open.*

eAppendix 6. Individual Risk of Bias Ratings

For individual risk of bias ratings per study please see the materials in the Risk of Bias folder provided on the open science framework https://osf.io/u3vdn/. Material include a) a data set including the study wise ratings, and b) a figure summarizing the ratings for each study.

© 2024 Terhorst Y et al. *JAMA Network Open.*

eAppendix 7. Risk of Bias and Study Quality: Sensitivity Analysis

To investigate the potential impact of the risk of bias and study quality, we conducted additional sensitivity analyses on the risk of bias (i.e., overall risk of bias as a sum across all risk of bias items and item-wise analysis comparing low and high risk of bias studies) and the impact of ITT analysis (i.e., per protocol compared to intention-to-treat studies). Please see the statistical analysis section in the manuscript for the general analysis methodology. Risk of bias was analyzed analogously to the sensitivity analysis described for participant, intervention, and study design characteristics. For details on the methodology regarding the analysis of the effects of IMI, please see eMethods 4. Results on HTE and study quality are presented in eAppendix 7, Table 1. Results on the effects and study quality can be found in eAppendix 7, Table 2.

© 2024 Terhorst Y et al. *JAMA Network Open.*

eAppendix 7. Extended Results

Table 1 – Influence of Study Quality on HTE Meta-Analysis

Outcome	*k*[1]	Level 1 (95%-CrI)[2]	Level 2 (95%-CrI)[3]	Level 3 (95%-CrI)[4]
**Overall RoB**				
Overall quality	102		0.09 (0.04 to 0.13)	0.07 (0.01 to 0.12)
~ Intercept (μ)		0.04 (-0.14 to 0.22)		
~ quality (β)		-0.01 (-0.02 to 0.01)		
~ lnER (β)		0.02 (-0.15 to 0.19)		
**Item-wise RoB**				
Random Sequence Generation				
~ Low risk (μ)	97	-0.03 (-0.08 to 0.02)	0.09 (0.04 to 0.13)	0.07 (0.01 to 0.12)
~ High risk (β)		0.15 (-0.03 to 0.33)		
~ lnER (β)		-0.00 (-0.17 to 0.17)		
Allocation Concealment				
~ Low risk (μ)	82	-0.04 (-0.10 to 0.02)	0.10 (0.05 to 0.14)	0.07 (0.01 to 0.13)
~ High risk (β)		-0.02 (-0.19 to 0.16)		
~ lnER (β)		-0.03 (-0.22 to 0.16)		
Blinding of Participants and Personnel				
~ Low risk (μ)	85	-0.02 (-0.19 to 0.24)	0.10 (0.06 to 0.14)	0.07 (0.01 to 0.13)
~ High risk (β)		-0.04 (-0.26 to 0.18)		
~ lnER (β)		0.03 (-0.16 to 0.21)		
Blinding of Outcome Assessment				
~ Low risk (μ)	80	0.01 (-0.08 to 0.10)	0.11 (0.06 to 0.15)	0.07 (0.01 to 0.13)
~ High risk (β)		-0.03 (-0.12 to 0.05)		
~ lnER (β)		0.06 (-0.14 to 0.25)		
Incomplete Outcome Data				
~ Low risk (μ)	99	-0.02 (-0.07 to 0.04)	0.09 (0.04 to 0.13)	0.07 (0.01 to 0.12)
~ High risk (β)		-0.02 (-0.09 to 0.05)		
~ lnER (β)		0.03 (-0.15 to 0.20)		
Selective Reporting				
~ Low risk (μ)	76	-0.02 (-0.07 to 0.04)	0.10 (0.06 to 0.14)	0.05 (0.00 to 0.10)
~ High risk (β)		-0.18 (-0.47 to 0.10)		
~ lnER (β)		0.01 (-0.16 to 0.19)		
Other Sources				
~ Low risk (μ)	97	-0.01 (-0.07 to 0.04)	0.09 (0.05 to 0.13)	0.06 (0.01 to 0.11)
~ High risk (β)		-0.08 (-0.23 to 0.06)		
~ lnER (β)		0.02 (-0.15 to 0.19)		
**Intention-to-treat vs. Per Protocol**				
Influence of Analysis Principle				
~ Per Protocol (μ)	102	-0.04 (-0.16 to 0.08)	0.09 (0.05 to 0.13)	0.07 (0.01 to 0.12)
~ Intention to Treat (β)		0.02 (-0.10 to 0.14)		
~ lnER (β)		0.02 (-0.57 to 0.19)		

Note. 1) Number of included studies. 2) Level 1 estimates in subgroup analysis quantify the HTE (lnVR $\hat{\mu}$; zero indicates equivalent variances in IG and CG) and present the influence of the investigated variables on HTE ($\hat{\beta}$; zero indicates no effect on HTE). *3) Level 2 estimates quantify how much estimates vary within studies. 4) Level 3 estimates quantify the extent estimates vary between studies. 95%-CrI quantify the 95% interval in which the true estimate lies, given the provided data.*

© 2024 Terhorst Y et al. *JAMA Network Open.*

eAppendix 7, Table 2 – Influence of Study Quality on Effects Meta-Analysis

Outcome	*k*[1]	Level 1 (95%-CrI)[2]	Level 2 (95%-CrI)[3]	Level 3 (95%-CrI)[4]
**Overall RoB**				
Overall quality	102		0.20 (0.13 to 0.28)	0.44 (0.35 to 0.55)
~ Intercept (μ)		-0.56 (-0.66 to -0.55)		
~ quality (β)		-0.04 (-0.15 to 0.07)		
**Item-wise RoB**				
Random Sequence Generation				
~ Low risk (μ)	97	-0.57 (-0.68 to -0.47)	0.20 (0.13 to 0.28)	0.45 (0.36 to 0.57)
~ High risk (β)		0.21 (-0.35 to 0.79)		
Allocation Concealment				
~ Low risk (μ)	82	-0.55 (-0.68 to -0.44)	0.23 (0.15 to 0.33)	0.46 (0.35 to 0.59)
~ High risk (β)		-0.02 (-0.61 to 0.55)		
Blinding of Participants and Personnel				
~ Low risk (μ)	85	-0.30 (-1.13 to 0.56)	0.24 (0.16 to 0.33)	0.47 (0.36 to 0.59)
~ High risk (β)		-0.29 (-1.15 to 0.54)		
Blinding of Outcome Assessment				
~ Low risk (μ)	80	-0.51 (-0.73 to -0.30)	0.22 (0.15 to 0.30)	0.36 (0.28 to 0.45)
~ High risk (β)		-0.07 (-0.31 to 0.17)		
Incomplete Outcome Data				
~ Low risk (μ)	99	-0.55 (-0.66 to -0.43)	0.20 (0.13 to 0.28)	0.44 (0.35 to 0.55)
~ High risk (β)		-0.01 (-0.25 to 0.22)		
Selective Reporting				
~ Low risk (μ)	76	-0.50 (-0.60 to -0.40)	0.22 (0.15 to 0.31)	0.35 (0.27 to 0.45)
~ High risk (β)		-0.27 (-1.04 to 0.46)		
Other Sources				
~ Low risk (μ)	97	-0.57 (-0.67 to -0.46)	0.21 (0.14 to 0.29)	0.44 (0.34 to 0.55)
~ High risk (β)		0.35 (-0.18 to 0.90)		
**Intention-to-treat vs. Per Protocol**				
Influence of Analysis Principle				
~ Per Protocol (μ)	102	-0.49 (-0.83 to -0.15)	0.20 (0.13 to 0.28)	0.44 (0.35 to 0.55)
~ Intention to Treat (β)		-0.07 (-0.42 to 0.28)		

Note. 1) Number of included studies. 2) Level 1 estimates in subgroup analysis quantify the effect (Hedges' g $\hat{\mu}$; zero indicates equivalent mean severity in IG and CG) and the influence of the investigated variables on the effects ($\hat{\beta}$; zero indicates no effect on Hedges' g). *3) Level 2 estimates quantify how much estimates vary within studies. 4) Level 3 estimates quantify the extent estimates vary between studies. 95%-CrI quantify the 95% interval in which the true estimate lies, given the provided data.*

© 2024 Terhorst Y et al. *JAMA Network Open.*

eAppendix 8 – Extended HTE Analysis Results

Bayesian Three-Level Meta-Regression Results for HTE in IMI for Depression.

Outcome	Level I estimates $\hat{\mu}$, (95%-CrI)	$\hat{\beta}_{lnER}$, (95%-CrI	Level II estimate sd, (95%-CrI)	Level III estimate sd, (95%-CrI)
**Primary outcome**				
Post-treatment	-0.02 (-0.07 to 0.03)	0.02 (-0.14 to 0.19)	0.09 (0.05 to 0.13)	0.07 (0.01 to 0.12)
**Study design characteristics**				
Setting				
Effectiveness	-0.05 (-0.14 to 0.04)	-0.10 (-0.60 to 0.39)	0.04 (0.00 to 0.11)	0.10 (0.02 to 0.16)
Efficacy	0.00 (-0.07 to 0.07)	0.08 (-0.12 to 0.29)	0.10 (0.06 to 0.14)	0.07 (0.01 to 0.13)
Control type				
WLC	0.06 (-0.06 to 0.16)	0.16 (-0.11 to 0.43)	0.13 (0.09 to 0.18)	0.06 (0.00 to 0.13)
TAU	-0.02 (-0.11 to 0.07)	0.25 (-0.29 to 0.81	0.05 (0.00 to 0.12)	0.09 (0.01 to 0.16)
Attention CG	-0.05 (-0.14 to 0.04)	-0.01 (-0.45 to 0.47)	0.03 (0.00 to 0.10)	0.07 (0.01 to 0.14)
Long-term HTE				
continuous		0.10 (-0.03 to 0.23)	0.08 (0.05 to 0.10)	0.08 (0.05 to 0.11)
~ Intercept (μ)	0.00 (-0.05 to 0.05)			
~ linear (β)	-0.00 (-0.01 to 0.01)			
~ quadratic (β)	-0.00 (-0.00 to 0.00)			
< 6 months	-0.00 (-0.05 to 0.04)	0.10 (-0.04 to 0.23)	0.08 (0.06 to 0.11)	0.09 (0.05 to 0.12)
6 months to 12 months	-0.00 (-0.10 to 0.10)	0.46 (-0.21 to 1.22)	0.03 (0.00 to 0.07)	0.03 (0.00 to 0.08)
1 year +	-0.02 (-0.16 to 0.14)	0.43 (-0.49 to 1.52)	0.03 (0.00 to 0.09)	0.06 (0.00 to 0.13
**Intervention characteristics**				
Guidance				
Guided	0.02 (-0.07 to 0.11)	0.12 (-0.14 to 0.38)	0.10 (0.04 to 0.15)	0.08 (0.01 to 0.14)
Technical guided	-0.07 (-0.16 to 0.02)	-0.14 (-0.43 to 0.14)	0.09 (0.02 to 0.15)	0.06 (0.00 to 0.13)
Unguided	-0.01 (-0.12 to 0.10)	0.08 (-0.37 to 0.54)	0.05 (0.00 to 0.14)	0.11 (0.01 to 0.19)
Therapeutic background				
CBT	-0.01 (-0.08 to 0.05)	0.07 (-0.19 to 0.32)	0.05 (0.00 to 0.10)	0.10 (0.03 to 0.14)
Third wave	-0.02 (-0.23 to 0.19)	0.25 (-0.48 to 0.99)	0.06 (0.00 to 0.18)	0.10 (0.01 to 0.23)
Technology				
Internet-based	-0.03 (-0.08 to 0.03)	-0.01 (-0.19 to 0.17)	0.09 (0.05 to 0.13)	0.07 (0.01 to 0.12)
Recent IMI				
Last decade (≥2013)	-0.01 (-0.07 to 0.04)	0.05 (-0.14 to 0.24)	0.10 (0.06 to 0.14)	0.07 (0.01 to 0.13)
Last five (≥2018)	-0.03 (-0.13 to 0.06)	0.07 (-0.19 to 0.33)	0.14 (0.09 to 0.19)	0.07 (0.00 to 0.16)
**Participant characteristics**				
Age		0.00 (-0.18to 0.19)	0.10 (0.06 to 0.14)	0.07 (0.01 to 0.12)
~ Intercept (μ)	-0.03 (-0.09 to 0.03)			
~ age (β)	0.01 (-0.02 to 0.04)			
Gender		0.01 (-0.17 to 0.18)	0.09 (0.04 to 0.13)	0.07 (0.01 to 0.12)
~ Intercept (μ)	-0.02 (-0.08 to 0.03)			
~ % female (β)	0.02 (-0.01 to 0.04)			
Baseline severity		0.10 (-0.07 to 0.28)	0.09 (0.04 to 0.12)	0.07 (0.01 to 0.11)
~ Intercept (μ)	0.00 (-0.05 to 0.06)			
~ severity (β)	0.04 (0.01 to 0.07)			

Note. Level 1 estimates in primary outcome and subgroup analysis quantify the HTE (lnVR $\hat{\mu}$; zero indicates equivalent variances in IG and CG). Regression results present the influence of the investigated variables on HTE ($\hat{\beta}$; zero indicates no effect on HTE). *Level 2 estimates quantify how much estimates vary within studies. Level 3 estimates quantify the extent estimates vary between studies. 95%-CrI quantify the 95% interval in which the true estimate lies, given the provided data.*

© 2024 Terhorst Y et al. *JAMA Network Open.*

eAppendix 9. Efficacy and Effectiveness Analysis Results

Results

Secondary analyses of the post-treatment effect size (ES) of IMI for depression showed a medium ES of *g*=-0.56 (95%-CrI: -0.46 to -0.66) favouring IMI. Between outcome (level II: 0.20, 95%-CrI: 0.13 to 0.28) and studies variance was high (level III: 0.44, 95%-CrI: 0.35 to 0.54).

Subgroup analyses showed a strong influence of study design characteristics: ES in efficacy studies (*g*=-0.64, 95%-CrI: -0.52 to -0.76) was twice as high as in effectiveness studies (*g*=-0.30, 95%-CrI: -0.16 to -0.43; Δ*g*=-0.33, 95%-CrI: -0.10 to -0.56). ES comparing IMI against waitlist-control groups was *g*=-0.76 (95%-CrI: -0.63 to -0.89), against attention-control groups *g*=-0.48 (95%-CrI: -0.12 to -0.84), and against treatment as usual *g*=-0.31 (95%-CrI: -0.20 to -0.44). The difference between waitlist-control group and attention-control was Δ*g*=-0.25 (95%-CrI: 0.01 to -0.50) and Δ*g*=-0.38 (95%-CrI: -0.19 to -0.57) compared to treatment-as-usual. ES for CBT-based IMI was (*g*=-0.55, 95%-CrI: -0.43 to -0.68) and for third-wave-based IMI (*g*=-0.48, 95%-CrI: -0.29 to -0.68). Regarding the underlying technology we found an average effect of *g*=-0.54 (95%-CrI: -0.45 to -0.64) for Internet-based interventions. Additional, subgroup analyses for app-based or computer-based interventions were not feasible due to an insufficient number of included studies.

Regarding the influence of guidance, we found the highest ES for guided IMI (*g*=-0.62, 95%-CrI: -0.50 to -0.75), followed by unguided IMI (*g*=-0.57, 95%-CrI: -0.24 to -0.91), and technical guided IMI (*g*=-0.31, 95%-CrI: -0.20 to -0.44). While favouring guided IMI, the difference to unguided IMI was non-significant overall (Δ*g*=0.07, 95%-CrI: -0.17 to 0.31), in efficacy studies (Δ*g*=0.09, 95%-CrI: -0.23 to 0.41), and effectiveness studies (Δ*g*=0.01, 95%-CrI: -0.25 to 0.27).

From participant characteristics, only baseline severity was affecting ES (β=-0.26, 95%-CrI: -0.17 to -0.36). Including the main and interactions effects of baseline severity and therapeutic guidance compared to unguided yielded a significant interaction effect between guidance and baseline severity, showing an increased impact of therapeutic guidance with increasing baseline severity

(interaction effect: $\hat{\beta}$=-0.24, 95%-CrI: -0.03 to -0.46). A similar tendency was observed for technical guidance compared to unguided (interaction effect: $\hat{\beta}$=-0.24, 95%-CrI: -0.48 to 0.00).

We did not find a robust linear or quadratic time effect on ES after baseline, but subgroup analysis showed the variation of an ES of *g*=-0.54 (-0.44 to -0.63) in comparisons within the first six months after baseline, *g*=-0.18 (-0.10 to -0.25) between six to twelve months, and *g*=-0.21 (-0.12 to -0.30) after one year or more. For a summary of all subgroup analyses on the ES see eAppendix 11, Table 1.

eAppendix 9, Table 1

Bayesian Three-Level Meta-Regression Results for ES of IMI for Depression.

Outcomes	k	Level I estimate (95%-CrI)	Level II estimate (95%-CrI)	Level III estimate (95%-CrI)
**Main effect**				
Post-treatment ES	102	-0.56 (-0.46 to -0.66)	0.20 (0.13 to 0.28)	0.44 (0.35 to 0.54)
**Study design characteristics**				
Setting				
Effectiveness	24	-0.30 (-0.16 to -0.43)	0.04 (0.00 to 0.14)	0.30 (0.20 to 0.43)
Efficacy	78	-0.64 (-0.52 to -0.76)	0.24 (0.17 to 0.34)	0.45 (0.34 to 0.58)
Type of control group				
Wait-list control	47	-0.76 (-0.63 to -0.89)	0.29 (0.20 to 0.40)	0.33 (0.21 to 0.45)
Treatment as usual	27	-0.31 (-0.20 to -0.44)	0.06 (0.00 to 0.20)	0.26 (0.15 to 0.40)
Attention control	24	-0.48 (-0.12 to -0.84)	0.04 (0.00 to 0.15)	0.86 (0.58 to 1.23)
**Intervention characteristics**				
Guidance				
Guided IMI	56	-0.62 (-0.50 to -0.75)	0.18 (0.06 to 0.29)	0.41 (0.30 to 0.53)
Technical guided IMI	27	-0.50 (-0.32 to -0.68)	0.28 (0.16 to 0.45)	0.37 (0.20 to 0.55)
Unguided IMI	25	-0.57 (-0.24 to -0.91)	0.12 (0.00 to 0.48)	0.81 (0.50 to 1.18)
Therapeutic background				
CBT	71	-0.55 (-0.43 to -0.68)	0.04 (0.00 to 0.10)	0.50 (0.39 to 0.63)
Third wave	14	-0.48 (-0.29 to -0.68)	0.16 (0.01 to 0.39)	0.24 (0.02 to 0.50)
Technology				
Internet-based	88	-0.54 (-0.45 to -0.65)	0.19 (0.13 to 0.26)	0.37 (0.29 to 0.45)
Recent IMI				
Last decade (≥2013)	82	-0.56 (-0.44 to -0.69)	0.25 (0.16 to 0.35)	0.48 (0.36 to 0.61)
Last five (≥2018)	39	-0.63 (-0.41 to -0.86)	0.33 (0.22 to 0.51)	0.63 (0.40 to 0.88)
Long-term ES				
continuous	102		0.17 (0.14 to 0.21)	0.45 (0.37 to 0.54)
~ Intercept (μ)		-0.55 (-0.45 to -0.66)		
~ linear time (β)		0.01 (-0.01 to 0.02)		
~ quadratic time (β)		-0.00 (-0.00 to 0.00)		
< 6 months	102	-0.54 (-0.44 to -0.63)	0.17 (0.13 to 0.21)	0.45 (0.37 to 0.54)
6 months to 12 months	21	-0.18 (-0.10 to -0.25)	0.05 (0.00 to 0.14)	0.12 (0.03 to 0.22)
1 year +	16	-0.21 (-0.12 to -0.30)	0.04 (0.00 to 0.10)	0.14 (0.07 to 0.23)

© 2024 Terhorst Y et al. *JAMA Network Open.*

Outcomes	k	Level I estimate<br>$\hat{\mu}/\hat{\beta}$, (95%-CrI)	Level II estimate<br>sd, (95%-CrI)	Level III estimate<br>sd, (95%-CrI)
**Participant characteristics**				
Age	94		0.22 (0.15 to 0.30)	0.43 (0.33 to 0.53)
~ Intercept (μ)		-0.56 (-0.46 to -0.66)		
~ age (β)		0.07 (-0.03 to 0.16)		
Gender	98		0.21 (0.14 to 0.28)	0.45 (0.35 to 0.56)
~ Intercept (μ)		-0.55 (-0.16 to -0.95)		
~ % female (β)		-0.01 (-0.55 to 0.54)		
Baseline severity	99		0.10 (0.01 to 0.19)	0.46 (0.37 to 0.57)
~ Intercept (μ)		-0.58 (-0.48 to -0.68)		
~ baseline severity (β)		-0.26 (-0.17 to -0.35)		
**Interactions**				
Baseline severity X guidance	76		0.06 (0.00 to 0.16)	0.53 (0.42 to 0.67)
(UG vs. GS)		-0.54 (-0.34 to -0.76)		
~ Intercept (μ)		-0.06 (-0.24 to 0.12)		
~ baseline severity (β)		-0.10 (-0.33 to 0.14)		
~ guidance (UG=0, GS=1) (β)		-0.24 (-0.03 to -0.46)		
~ interaction (β)				
Baseline severity X guidance	67		0.09 (0.00 to 0.23)	0.58 (0.42 to 0.78)
(UG vs. TG)		-0.52 (-0.28 to -0.77)		
~ Intercept (μ)		-0.16 (-0.39 to 0.07)		
~ baseline severity (β)		-0.04 (-0.34 to 0.25)		
~ guidance (UG=0, TG=1) (β)		-0.24 (-0.48 to 0.00)		
~ interaction (β)				

Note. Level 1 estimates in subgroup analysis quantify the effect (Hedges' g $\hat{\mu}$; zero indicates equivalent mean severity in IG and CG) and the influence of the investigated variables on the effects ($\hat{\beta}$; zero indicates no effect on Hedges' g)*. Level 2 estimates quantify how much estimates vary within studies. Level 3 estimates quantify the extent estimates vary between studies. 95%-CrI quantify the 95% interval in which the true estimate lies, given the provided data.*

© 2024 Terhorst Y et al. *JAMA Network Open.*
