# Supplementary material for: Heterogeneity of Treatment Effects in Internet- and Mobile-Based Interventions for Depression: A Systematic Review and Meta-Analysis
Source: JAMA Netw Open. 2024 Jul 18;7(7):e2423241. doi: 10.1001/jamanetworkopen.2024.23241 (PMC11258589; doi:10.1001/jamanetworkopen.2024.23241)
Supplement: Supplement 3. — Data Sharing Statement [file jamanetwopen-e2423241-s003.pdf]

Data Sharing Statement

Terhorst. Heterogeneity of Treatment Effects in Internet- and Mobile-Based Interventions for Depression. *JAMA Netw Open*. Published July 18, 2024.
doi:10.1001/jamanetworkopen.2024.23241

Data
Data available: Yes
Data types: Data (not involving human participants)
How to access data: Analysis code and used data are freely available in the OSF project: https://osf.io/u3vdn/
When available: beginning date: 12-22-2023

Supporting Documents
Document types: Statistical/analytic code
How to access documents: Analysis code and used data are freely available in the OSF project: https://osf.io/u3vdn/
When available: beginning date: 12-22-2023

Additional Information
Who can access the data: The dataset of the present meta-analysis and the analysis script is available at the open science framework under a CC-By Attribution 4.0 International license: https://osf.io/u3vdn/. Additional information can be provided by the corresponding author (YT) upon reasonable request. Support from the corresponding author depends on available resources.
Types of analyses: Meta-analysis code
Mechanisms of data availability: The dataset of the present meta-analysis and the analysis script is available at the open science framework under a CC-By Attribution 4.0 International license: https://osf.io/u3vdn/. Additional information can be provided by the corresponding author (YT) upon reasonable request. Support from the corresponding author depends on available resources.
